# Trypanosomiasis of camels (*Camelus dromedarius*) in Algeria: First report

**Published:** 2013

**Authors:** Omar Bennoune, Nezar Adili, Khaled Amri, Lakhdar Bennecib, Ammar Ayachi

**Affiliations:** 1 *Laboratory of Health, Animal Production and Environment, Department of Veterinary Medicine, Institute of Veterinary and Agricultural Sciences, University of Batna, Batna, Algeria;*; 2 *Department of Veterinary Medicine, Institute of Veterinary and Agricultural Sciences, University of Batna, Batna, Algeria.*

**Keywords:** Blood smears, Camel, Parasite, Trypanosomiasis

## Abstract

Camel trypanosomosis is a life-threatening disease in the camel species and responsible for severe economic losses either in milk or meat productions. This study was carried out on the south-east area of Algeria on 100 camels of various ages and either sex from two herds. Microscopic examination of blood smears revealed higher levels of trypanosomosis caused by *Trypanosoma* *evansi*, an elongated parasite with a kinetoplast and a single nucleus located in its half-length and one flagellum with great heterogeneity. This first investigation reveals higher infection rate than those observed in other countries using blood smears, the trypanosomosis attack has reached an alarming level and the occurrence of trypanosomosis at this high level on blood smears is like "the tree that hides the forest" and make up a serious and potential danger both on animal and public health. Therefore, radical preventive and offensive drastic measures must be taken against this menacing disease at the critical points to prevent the economic losses and to avoid possible human transmission.

## Introduction

Camel trypanosomosis is caused by *Trypanosoma evansi*, a hemoflagellate parasite identified for the first time in India in 1880.^1^ The vectors are bloodsucking flies of Tabanidae.^2^ Camel trypanosomosis is one of the main causes of camel infectious abortion in the Middle East and Africa.^3^ In Canary Islands, *T. evansi* is behind an outbreak of abortions and high neonatal mortality observed in camels.^4^ In males, camel trypanosomosis may cause severe irreversible testicular degeneration.^5^ This illness occur in the horses, the dogs as well as the elephants in Asia.^6^ Human cases of trypanosomosis caused by *T. evansi* has been reported in India.^7^^,^^8^ The trypanosomosis is responsible for significant reductions in the number of red blood cells (RBC), hemoglobin and packed cell volume (PCV).^9^

In Morocco, an epidemiological study revealed that seroprevalence of the trypanosomosis is 14.10% using card agglutination test for trypanosomiasis (CATT) and 18.20% using ELISA (Ab-ELISA).^10^ Recent outbreaks of trypanosomosis have been reported in Europe, Spain (Mainland),^11^ and France.^12^ So far, little information is available about camel diseases in Algeria. In these regions, a huge number of camels (*Camelus dromedarius*) are concentrated on the dry desert south areas of the country and constitute the major source of animal protein for nomads. Screening and detection of these hemoparasites helps initiate early diagnosis and instauration of appropriate actions and guarantee higher performance.

## Materials and Methods

**Animals and blood smears.** This study was carried out on two herds od *Camelus dromedarius* from the south-east region of Algeria. The region harbors more than 50% of camels in Algeria.^13^ Some of these animals are apparently in good health while others are cachectic and weak and cases of abortions have been occurred in these herds. The samples were taken from 100 dromedaries, 51 males and 49 females of different ages.

Blood samples have been carefully taken after animal immobilization. These samples were easily drawn from the jugular vein and especially when moderate pressure was applied on the way of the vein to mid-distance between the thoracic inlet and the head. Disposable sterile apyrogenic syringes (C.I. Cretes, Alger, Algeria) have been used after disinfection of the site of blood sampling. 

The blood smears were achieved directly after blood collection without anticoagulants which may interfere and induce some cytoplasmic and morphometric cell changes and on the extreme provoke degranulation of some blood cells. The blood smears were quickly dried and fixed by the methanol (Prolabo, Paris, France) to avoid all possible deteriorations then stained in the laboratory by the May Grünwald Giemsa (MGG, Riedel de Haen, Seelze, Germany).

**Microscopic** **examination** **and** **measurements. **Stained blood smears were observed using standard optic microscope (Axioskop 20; Carl zeiss, Göttingen, Germany), the zone of examining of the blood smears was located close to the tail where the thickness was ideal and suitable for the best observation and photographs, parasite measurements were done using an ocular micrometer.

## Results

**Fig 1:**
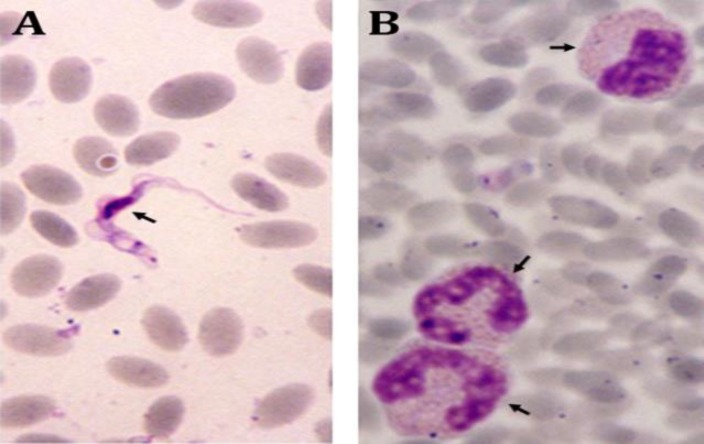
**A.** *Trypanosoma* with a well visible kinetoplast (arrow), nucleus and flagellum with elliptic RBC; **B.** Three eosinophils with variable forms of nucleus (arrows), (MGG, 1000×).

After careful examination of the blood smears and identification of the formed blood elements, trypanosomes were observed on 14 blood smears (9 males and 5 females) in different stages. The parasites were very well visible with a spindle shaped stretched out body with one nucleus situated in its half-length and one flagellum (Fig. 1). About 14.00% of camels of the two herds were affected and constituted a source of infection for other herds in this region.

The infection has reached an alarming rate in this area and a rate of 14.00% on blood smears needs careful attention and rapid actions and the real infection rate may spike greatly with ultrasensitive methods. Affected dromedaries showed a marked eosinophilia with three eosinophils per field of observation that is a natural defense mechanism against parasites (Fig. 1). The measurement of the parasite by ocular micrometer showed great heterogeneity (14.27 ± 6.36 µm) and the nucleus reached the length of 3.40 µm. The size of these blood parasites far exceeds the resolving power of ordinary light microscopes and readily observed even at lower magnifications.

## Discussion

The rate of infection is higher than that observed in some neighboring countries; the prevalence of trypanosomosis in Mauritania is only 1.30% on blood smears.^14^ The prevalence of *T. evansi* infection in Mauritania increased highly and reached 16.20% and 25.20% using Card agglutination test for trypanosomiasis (CATT) and Immunofluorescence antibody test (IFAT), respectively.^14^

In the Canary Islands, the prevalence of trypanosomosis is least and a rate of 0.94% is observed by microscopic examination, but increases considerably by the use of ultrasensitive techniques and 4.83% was positive using CATT/*T. evansi*.^15^ Another study in the Canary Islands revealed a seroprevalence of 9.00% using an indirect enzyme immunoassay (Ab-ELISA) and is higher (1.30%) than that observed by microscopic examination.^16^

In India, the stained blood smears revealed 7.50% infestation, whereas the number of seropositive camels using ELISA technique was four times higher than (31.66%) the number observed by microscopic examination.^17^ The same was observed in Somalia, where the rate of infestation was 1.70% by microscopic examination but soared up to 56.40% by the use of more sensitive techniques like micro-ELISA.^18^ Thus, observation of these hemoparasites in blood smears involves systematically the use of more sensitive techniques to estimate the real extent of this disease and to act appropriately and correctly.

Blood smears, require reduced material and skills and continue to be a convenient and useful diagnostic method for direct observation of these hemoflagellate parasites and are mostly used by veterinarians and still a confirmative tool and one of the best techniques employed for studying the parasite but less sensitive than other techniques such as the ELISA and the agglutination tests.

The use of appropriate rationale preventive and medical treatments by veterinarians is the key element in the control of trypanosomosis and preventing emergence of drug resistance like that observed in some Chinese *T. evansi* isolates.^19^ Recently, some derivative compounds from *Azadirachta indica* have been reported to have trypano-prophylactic effect and may be used against *T. evansi*.^20^ The transhumance by seasonal movement of livestock has profound effect on the epidemiology and spread of this disease in Africa.^21^ Due to the complicated transmission factors, survey and control of this disease involves complete contribution of competent territorial authorities.

## References

[1] Radostits OM, Blood DC, Gay CC (1997). Veterinary medicine: A textbook of diseases of cattle, sheep, pig, goat and horse.

[2] Fallis AM (1980). Arthropods as pests and vectors of disease. Vet Parasitol.

[3] Tibary A, Fite C, Anouassi A, Sghiri A (2006). Infectious causes of reproductive loss in Camelids. Theriogenology.

[4] Gutierrez C, Corbera JA, Juste MC (2005). An outbreak of abortions and high neonatal mortality associated with Trypanosoma evansi infection in dromedary camels in the Canary Islands. Vet Parasitol.

[5] Al-Qarawi AA, Omer HS, Abdel-Rahman HA (2004). Trypanosomiasis-induced infertility in dromedary (Camelus dromedarius) bulls: Changes in plasma steroids concentration and semen characteristics. Anim Reprod Sci.

[6] Bowman DD ( 1995). Georgis’ parasitology for veterinarians.

[7] Prashant PJ, Vijay RS, Rajaram MP (2005). Human trypanosomiasis caused by Trypanosoma evansi in India: The first case report. Am J Trop Med Hyg.

[8] Powar RM, Shegokar VR, Joshi PP (2006). A rare case of human trypanosomiasis caused by Trypanosoma evansi. Indian J Med Microbiol.

[9] Chaudhary ZI, Iqbal J (2000). Incidence, biochemical and hematological alterations induced by natural trypanosomosis in racing dromedary camels. Acta Trop.

[10] Atarhouch T, Rami M, Bendahman MN (2003). Camel trypanosomosis in Morocco 1: Results of a first epidemiological survey. Vet Parasitol.

[11] Tamarit A, Gutierrez C, Arroyo R (2010). Trypanosoma evansi infection in mainland Spain. Vet Parasitol.

[12] Desquesnes M, Bossard G, Patrel D (2008). First outbreak of Trypanosoma evansi in camels in metropolitan France. Vet Rec.

[13] Ben Aissa R, Tisserand JL (1989). The dromedary in Algeria. Seminar on the digestion, nutrition and feeding camel [French].

[14] Dia ML, Diop C, Aminetou M (1997). Some factors affecting the prevalence of Trypanosoma evansi in camels in Mauritania. Vet Parasitol.

[15] Gutierrez C, Juste MC, Corbera JA (2000). Camel trypanosomosis in the Canary Islands: Assessment of seroprevalence and infection rates using the card agglutination test (CATT/T evansi) and parasite detection tests.. Vet Parasitol.

[16] Molina JM, Ruiz A, Juste MC (2000). Seroprevalence of Trypanosoma evansi in dromedaries (Camelus dromedarius) from the Canary Islands (Spain) using an antibody Ab-ELISA. Prev Vet Med.

[17] Pathak KM, Arora JK, Kapoor M (1993). Camel trypanosomosis in Rajasthan, India. Vet Parasitol.

[18] Baumann MP, Zessin KH (1992). Productivity and health of camels (Camelus dromedarius) in Somalia: Associations with trypanosomosis and brucellosis. Trop Anim Health Prod.

[19] Zhou J, Shen J, Liao D (2004). Resistance to drug by different isolates Trypanosoma evansi in China. Acta Trop.

[20] Habila N, Humphrey NC, Abel AS (2011). Trypanocidal potentials of Azadirachta indica seeds against Trypanosoma evansi. Vet Parasitol.

[21] Macpherson CNL (1995). The effect of transhumance on the epidemiology of animal diseases. Prev Vet Med.

